# Anti-Amyloid Therapies for Alzheimer’s Disease and the Amyloid Cascade Hypothesis

**DOI:** 10.3390/ijms241914499

**Published:** 2023-09-24

**Authors:** Ernesto Fedele

**Affiliations:** 1 Pharmacology and Toxicology Unit, Department of Pharmacy, School of Medical and Pharmaceutical Sciences, University of Genoa, Viale Cembrano 4, 16148 Genoa, Italy; ernesto.fedele@unige.it; 2 IRCCS Ospedale Policlinico San Martino, 16132 Genoa, Italy

**Keywords:** Alzheimer’s disease, Aβ antibodies, amyloid cascade hypothesis, clinical trials

## Abstract

Over the past 30 years, the majority of (pre)clinical efforts to find an effective therapy for Alzheimer’s disease (AD) focused on clearing the β-amyloid peptide (Aβ) from the brain since, according to the amyloid cascade hypothesis, the peptide was (and it is still considered by many) the pathogenic determinant of this neurodegenerative disorder. However, as reviewed in this article, results from the numerous clinical trials that have tested anti-Aβ therapies to date indicate that this peptide plays a minor role in the pathogenesis of AD. Indeed, even Aducanumab and Lecanemab, the two antibodies recently approved by the FDA for AD therapy, as well as Donanemab showed limited efficacy on cognitive parameters in phase III clinical trials, despite their capability of markedly lowering Aβ brain load. Furthermore, preclinical evidence demonstrates that Aβ possesses several physiological functions, including memory formation, suggesting that AD may in part be due to a loss of function of this peptide. Finally, it is generally accepted that AD could be the result of many molecular dysfunctions, and therefore, if we keep chasing only Aβ, it means that we cannot see the forest for the trees.

## 1. Alzheimer’s Disease

Alzheimer’s disease (AD) is the neurodegenerative disorder responsible for approximately 60–70% of all cases of dementia, which affects more that 55 million people worldwide and is predicted to reach 152 million by 2050, assuming that prevalence will remain constant [1]. The main neuropathological features of AD are represented by extracellular deposits of Aβ, known as plaques, and aggregates of hyperphosphorylated tau, known as neurofibrillary tangles (NTFs), inside neurons. The early clinical sign of AD is represented by a decline in the capacity of remembering recent events, but with the progression of the disease, more symptoms manifest (e.g., confusion, disorientation, mood changes, memory loss, cognitive alterations, increasing difficulties in writing, reading, speaking, etc.) and become more and more severe over time, thus dramatically affecting patients’ daily life [2].

Two main forms of the disease can be distinguished based on the age onset. Thus, late onset AD (LOAD) manifests at an age older than 65 years and accounts for approximately 95% of all AD cases, whereas early onset AD (EOAD) shows an age of onset ranging from 35 to 65 years and represents 1–6% of all cases [3,4]. Moreover, AD can be also classified from a genetic point of view into sporadic (SAD) and familial (FAD) forms [3,4]. SAD shows no familial aggregation and is considered to result from a complex combination of genetic variants, environmental and lifestyle risk factors, as well as comorbidities. FAD is a rare form of the disease (1–2% of all AD patients), also known as ADAD (autosomal dominant AD) or DIAD (dominantly inherited AD), caused by mutations of the amyloid precursor protein (*APP*) gene or of the *PSEN1/2* genes (see below). Most of sporadic cases are LOAD, while familial AD predominantly presents with early onset.

Among the risk factors for AD, age is considered the most important one, with an estimated prevalence increase of 19% in persons aged 75–84 years and up to 50% for those older than 85 years. Apolipoprotein E (*APOE*) gene variants follow next, as one copy of the ε4 allele can increase the risk of developing AD by 2–6 times, while two copies increase the risk by 7–21 times. Genetic studies have identified many other AD susceptibility loci and a large number of rare variants associated to AD (e.g., TREM 2, SORL1, ABCA7) [3,5,6,7]. Moreover, environmental factors (e.g., heavy metals, pesticides), comorbidities (e.g., diabetes, traumatic brain injury) and lifestyle (e.g., smoking, high-fat diet) can impact AD risk [5,8]. Finally, growing evidence suggest that dysbiosis of the gut microbiome can be involved in the pathogenesis of AD by dysregulating the gut–brain axis [9,10].

## 2. The Amyloid Cascade Hypothesis: From Plaques to Soluble Oligomers

Since its discovery in the early 1980s [11], no endogenous molecule has been witch-hunting like Aβ. Indeed, following its characterisation as the peptide monomer forming the insoluble senile plaques in the brain of Alzheimer’s disease patients, researchers focused their attention almost exclusively on its toxic properties. In 1992, this view culminated in the formulation of the original version of the amyloid cascade hypothesis (ACH) by Hardy and Higgins [12]. Briefly, it postulated that the peptide deposition in brain parenchyma plaques is the trigger of a pathocascade of cellular events leading to synaptic loss and neurodegenerative processes responsible for extensive neuronal cell death and, consequently, AD dementia. The ACH soon became a dogma that has been driving the search for effective pharmacological and non-pharmacological therapies for AD in the last three decades. 

Studies to unveil the biochemical origin of Aβ led in a few years to the discovery of the transmembrane APP [13,14,15,16] and its different processing in the non-amyloidogenic and amyloidogenic pathways [17,18,19,20,21,22]. Basically, the key difference between the two pathways lies in the first enzymatic processing of APP. In the non-amyloidogenic pathway, APP is cleaved by α-secretase in the middle of the Aβ sequence, generating a soluble fragment alpha (sAPPα) and leaving the C-terminal fragment alpha (αCTF) in the membrane. On the contrary, in the amyloidogenic pathway, β-secretase cleaves APP at the N-terminus of the Aβ sequence, originating sAPPβ and transmembrane βCTF. Therefore, upon the subsequent action of γ-secretase, the cleavage of βCTF, but not that of αCTF, results in the production of Aβ [23,24]. The processing of βCTF by γ-secretase occurs at different sites, thus yielding many Aβ peptides of variable length, with Aβ_40_ being the most abundant form in the brain, followed by Aβ_38_ and Aβ_42_ [25]. Among these forms, Aβ_42_ was considered the culprit of AD since it is the major form deposited in senile plaques, in line with its higher hydrophobicity and propensity to self-aggregation. Moreover, it was initially thought that Aβ was not generated during physiological APP metabolism but occurred only under pathological conditions [26]. However, it soon became clear that this is not the case, since it was later found that Aβ is normally produced in the healthy brain, where it underlies important physiological processes (see below). 

The finding of mutations in the *APP* gene and in the genes of the two γ-secretase complex proteins PSEN1/2 in FAD, leading to an increased production of Aβ_42_ and alteration of the Aβ_42_/Aβ_40_ ratio, provided further support to the ACH. 

Yet, the evidence that the amount of Aβ plaques in the AD brain did not correlate either with disease severity or cognitive impairment, along with the observation that a highly significant percentage of cognitively unimpaired elderly people (up to 40%) showed abnormal amyloid plaque burden, led researchers to modify the original ACH. 

Thus, the current version of the hypothesis indicates that the accumulation of soluble Aβ oligomers is the upstream pathogenic process for AD that occurs over many years before clinical onset [27]. However, Aβ accumulation seems to occur through different mechanisms, being mainly due to increased production or decreased clearance in the familial and sporadic forms, respectively [27]. 

The amyloidocentric view of AD pathogenesis also predicts that Aβ accumulation is the causative factor of hyperphosphorylated tau (p-tau) aggregation and formation of neurofibrillary tangles, leading to neurodegeneration and cognitive deficits in a defined chronological order [28,29].

## 3. Is (Pre)Clinical Evidence in Favour of or against the Amyloid Cascade Hypothesis?

Scientific progress is based on the formulation of hypotheses that then need to be confirmed or disproved by using all the available means to gather experimental data, which, after being adequately validated, become scientific evidence in favour of or against the original premise. 

This section briefly summarises the main points regarding preclinical data of the ACH, and then focuses on the results reported over the last 20 years or so from the most relevant phase III clinical trials (or phase II if phase III is not available) that tested immunisation against Aβ on cognitive deterioration. The results of other Aβ-lowering therapeutic approaches with β- and γ-secretase inhibitors, though mostly negative, have not been considered, since they could be biased by the fact that these two enzymes can process a hundred endogenous substrates other than APP and CTFs.

### 3.1. Preclinical Evidence

A myriad of in vitro studies have reported that Aβ is cytotoxic in a variety of cell models by activating many molecular death-inducing cascades (e.g., calcium overload, mitochondrial toxicity, oxidative stress) through different cellular mechanisms (e.g., PrP^c^, i/mGluRs, RAGE, nAChRs). In addition, Aβ also induces a neuroinflammatory response by interacting with astrocytes and microglia, leading to the release of proinflammatory cytokines and chemokines that further exacerbate neuronal cell death [30,31,32,33]. 

However, it has been argued that many of these studies used concentrations of Aβ up to 1000 times higher than biological ones [34]. Nevertheless, the real concentration of the different forms of Aβ (monomers, dimers, oligomers) in the extracellular microenvironment of the AD brain can be difficult to assess and remains highly controversial [35].

On the contrary, what is not controversial is the observation that there is no widespread frank neuronal death, and no neurofibrillary tangles (NTFs) are present in the vast majority of transgenic murine models of FAD. This is observed in mice overexpressing human mutant APP (note that there is no evidence of APP overexpression in AD patients, except in very rare forms of early onset FAD [36]) or mutated hAPP/hPSEN1 that, on the other hand, produce high levels of Aβ [37,38]. NTFs are observed in transgenic mice when, in addition to mutated *hAPP* or *hAPP/hPSEN1* genes, they carry also the P301L human gene for protein tau (hMAPT), a mutation not associated with FAD [39,40]. However, NTFs, neurodegeneration, and memory deficits are also present in murine models with only the expression of the mutated *hMAPT* gene, indicating that Aβ does not seem necessary for tau-induced neuropathological alterations but can enhance them [39,41].

Finally, memory deficits are almost completely reversed in AD animal models upon genetic or pharmacological manipulations reducing Aβ levels, suggesting some sort of reversible damage, which does not occur in AD patients. 

Undoubtedly, however, in vivo experimental models of human pathologies, with their pros and cons, have the merit of providing the possibility for novel therapeutic approaches to enter the clinical trial phases, which have the final word on confirming or refuting the original working hypothesis. 

### 3.2. Clinical Evidence: Active Immunotherapies

The first immunotherapy for AD patients started with the active immunisation protocol using the AN1972 vaccine (aggregated human Aβ_1–42_) on a total of 372 (300 vaccine vs. 72 placebo) patients with mild to moderate AD in a randomised, placebo-controlled, double-blind, phase IIa trial [42]. However, the trial was stopped, as 6% of patients experienced meningoencephalitis, most of them (274) after two administrations. Analysis of the small antibody-responder group (59 patients) revealed no differences from placebo in cognitive, disability, and global change scores at 12 months (Alzheimer’s Disease Assessment Scale—cognitive subscale, ADAS-cog; Disability Assessment for Dementia, DAD; Clinical Dementia Rating Scale, CDR; Mini-Mental State Examination, MMSE; AD Cooperative Study—Clinical Global Impression of Change, ADCS-CGIC) [43]. Instead, the z-score composite of the nine-component Neuropsychological Test Battery (NTB) showed some reduced decline, which was reported to be maintained at 4.6 years in a smaller group of the original antibody-responder population (25 patients) [43]. Results of another 6-year follow-up study on AN1972 showed that progression to severe AD stages was not prevented in immunised patients, and there was no evidence of improved survival despite a reduction of the Aβ load [44].

CAD106 is a second-generation vaccine, comprising multiple copies of Aβ_1–6_ coupled to a bacteriophage Qb coat protein carrier, able to induce a consistent anti-Aβ immune response (approx. 74% responders) with a better safety profile in a 52-week, randomised, double-blind, placebo-controlled, first-in-human trial enrolling two cohorts of patients (58 total) with mild to moderate AD [45]. Free Aβ concentration in plasma decreased in immunised patients, suggesting binding of antibodies to the peptide. However, no significant differences were detected between immunised and unimmunised patients in CSF biomarkers (Aβ and p-tau), cognitive function assessment (MMSE; CDR; AD Cooperative Study—Activities of Daily Living, ADCS-ADL) and brain atrophy (volumetric MRI). 

Another study reported the results obtained with CAD106 in two phase IIa, 52-week, randomised, placebo-controlled trials (core study), followed by a 66-week open-label evaluation (extension study), on a total of 58 patients with mild AD [46]. In these trials, CAD106 induced a prolonged anti-Aβ immune response both in the core and extension studies, with 63.8% of patients considered responders. CAD106 was generally well tolerated, with mild or moderate adverse events consistent with earlier studies. Overall, however, no treatment-related effects were observed in the core studies on CSF biomarkers (Aβ_40/42_ and p-tau), on brain volume (MRI), and on cognitive assessment (MMSE; ADAS-Cog; Global Deterioration Scale, GDS). A decrease of CSF p-tau from the core study baseline was observed in the extension studies. 

Finally, two different doses of CAD106 (150 and 450 mg), with and without adjuvants, were trialled in a 90-week, randomised, double-blind, placebo-controlled phase IIb study on a total of 121 patients with mild AD, most of them (101/121) being *APOE ε4* carriers [47]. The administration of CAD106 was generally well tolerated, and most of adverse events were of mild to moderate severity. CAD106 induced a significant immune response in a dose-dependent manner, with the higher dose showing the higher increase of Aβ-IgG and higher frequency of responders (89.1%). Indeed, most responders (81.1%) were classified as strong serological responders (SSRs). An amyloid-PET exploratory analysis in a small subgroup of patients (11 SSRs, 2 non responders, and 2 placebos) showed a longitudinal decrease in amyloid-PET signal in SSRs but not in controls. Of note, the volumetric MRI results indicated an unexpected larger decrease in cortical grey matter in SSRs versus controls from baseline to week 78, although there was no correlation with the antibody response. Lastly, CAD106 did not show any significant longitudinal change in the exploratory assessment of cognitive effects (ADAS-cog, MMSE, ADCS-ADL, CDR) in comparison with the placebo. 

Recently, CAD106 has been included in one pivotal phase 2/3 study of 5–8-duration under the umbrella of the Alzheimer’s Prevention Initiative Generation Program. The study will assess vaccine safety and efficacy to slow progression or even prevent onset of AD in cognitively healthy subjects at high risk for the development of clinical AD, based on their age, APOE genetics, and elevation of brain amyloid [48].

A third vaccine, named ACC-001 (Vanutide Cridificar), consisting of Aβ_1–7_ peptides conjugated to a carrier protein, was tested on 245 patients with mild-to-moderate AD in two randomised, third-party-unblinded, placebo-controlled phase IIa trials with a multiple ascending-dose schedule (3, 10, 30 mg) [49]. Patients were treated with up to five doses of vaccine or placebo and followed for up to 12 months after last administration. The results indicated that, although ACC-001 had an acceptable safety profile and was able to evoke significant and sustained anti-Aβ IgG titers, it did not show differences for exploratory cognitive assessment, volumetric brain MRI measurements, and CSF biomarker analysis between the treatment and placebo groups.

### 3.3. Clinical Evidence: Passive Immunotherapies

The second approach to lower Aβ levels evaluated in clinical trials is passive immunisation with anti-Aβ antibodies.

#### 3.3.1. Bapineuzumab

Bapinezumab, a N-terminus (Aβ_1–5_)-directed antibody able to bind fibrillar, oligomeric, and monomeric forms of the peptide, was the first to be tested in a phase III development program consisting of four randomised, double-blind, placebo-controlled, phase III trials conducted in parallel on mild to moderate AD patients with (carriers) or without (non-carriers) the *APOE ε4* genotype (Table 1). In the first two trials (studies 301 and 302), the modified intention-to-treat population included 1090 carriers and 1114 non-carriers [50]. Bapineuzumab was intravenously (i.v.) administered at the dose of 0.5 mg/kg to 658 carriers and to 314 non-carriers, and at the dose of 1 mg/kg to 307 non-carriers, every 13 weeks (up to six infusions) for 78 weeks. Bapineuzumab was planned to be evaluated also at 2 mg/kg in non-carriers, but this dose was discontinued early in the trial due to a high rate of clinically symptomatic side effects (i.e., amyloid-related imaging abnormalities with effusion or oedema). All the 141 participants initially assigned to receive the 2.0 mg/kg dose were reassigned to the 1 mg/kg group and were included only in the safety analyses. In both trials, approximately 70% of patients in the bapineuzumab groups completed the study, and the antibody did not show any beneficial effect on cognitive coprimary (ADAS-cog11 and DAD) or other clinical endpoints (MMSE; CDR—Sum of Boxes, CDR-SB; NTB) with respect to placebo, except for a significant difference in the DAD score in a mild AD subpopulation (MMSE ≥ 20). As for target engagement, Aβ load, assessed by amyloid-PET, was almost unchanged at week 71 in the *APOE ε4* carrier group treated with bapineuzumab, whereas it increased in the placebo group, a finding confirmed in a subsequent analysis [51]. On the other hand, significant reductions in CSF p-tau concentration were observed in the carrier population and in the 1 mg/kg non-carrier group. Finally, the volumetric MRI analysis showed no significant effects of bapineuzumab on brain volume loss rate in both patient populations. 

In the other two trials (studies 3000 and 3001), which were prematurely terminated due to the negative results observed in the first two ones, 398 carriers and 102 non-carriers treated with 0.5 mg/kg bapineuzumab and 94 non-carriers treated with 1 mg/kg bapineuzumab completed the study [52]. Also in these trials, bapineuzumab failed to show significant effects for coprimary (ADAS-Cog11, DAD) and secondary efficacy (NTB, CDR-SB, DS) outcomes both in carriers and non-carriers, with the only exception of a significant 0.1 difference in favour of bapineuzumab (0.5 mg/kg) for non-carrier NTB total z-score. In addition, in subgroups of patients, no significant differences versus placebo were reported for amyloid-PET, CSF p-tau, and whole-brain volume loss.

Finally, two phase III extension studies of the 3000 and 3001 trials (3002 non-carriers and 3003 carriers, respectively) reported no significant changes between the dose groups in exploratory analysis for cognitive and functional outcomes, although interpretation of these results was limited by the absence of a placebo group in the extension period and the early discontinuation of the trials [53]. Analysis of CSF p-tau and of whole-brain volume on small groups of patients showed that bapineuzumab did not induce significant differences.

**Table 1:** Phase III trials testing bapineuzumab.

Study Design and Duration	Study Population	Cognitive Primary Endpoint	CSF or Plasma Aβ	Amyloid PET	Volumetric MRI
Phase III, randomised, double-blind, placebo-controlled 78 weeks [50,51]	Mild to moderate AD, *APOE ε4* carriers and non-carriers	ADAS-cog11 and DAD No significant effects in the whole population	ND	Significant effects in carriers	No significant effects
Phase III, randomised, double-blind, placebo-controlled (3000 and 3001 studies) 78 weeks Terminated early [52]	Mild to moderate AD, *APOE ε4* carriers and non-carriers	ADAS-cog11 and DAD No significant effects in the whole population	Significant increase in plasma	No significant effects	No significant effects
Phase III extension of 3000 and 3001 studies 208 weeks Terminated early [53]	Mild to moderate AD, *APOE ε4* carriers and non-carriers	ADAS-cog11, DAD, and MMSE No significant effects in the whole population	ND	Insufficient data	No significant effects

ND, not determined.

#### 3.3.2. Solanezumab

Solanezumab is a humanised immunoglobulin G1 monoclonal antibody recognising the mid-domain of soluble monomeric Aβ, which was tested in two randomised, double-blind, placebo-controlled, phase III trials on mild-to-moderate AD patients at the dose of 400 mg i.v., once every 4 weeks for 80 weeks (EXPEDITION 1 and 2) (Table 2) [54]. A total of 1027 patients in the two trials were assigned to receive the antibody, and 776 completed the study. Primary outcomes for efficacy analysis originally included ADAS-Cog11 and ADCS-ADL, whereas ADAS-Cog14, CDR-SB, MMSE, NPI (Neuropsychiatric Inventory), RUD-Lite scale (Resource Utilization in Dementia Lite), EQ-5D (the European Quality of Life 5 Dimensions scale) and QOL-AD (Quality of Life in Alzheimer’s Disease scale) were secondary outcomes. Patients were also APOE genotyped and were subjected to analysis of Aβ plasma levels, Aβ and tau CSF levels, MRI brain volumetric measures, and amyloid-PET imaging. In general, both trials did not reveal meaningful improvement in primary and secondary outcomes from baseline to week 80. The EXPEDITION 2 trial reported a significant change of 2.3 points in the ADCS-ADL score (range 0 to 78) only for patients with mild AD, a significant change of 0.8 points in the MMSE score (range 0 to 30) in the whole population, and a significant change of 1 point in the MMSE score for patients with moderate AD. With regards to biomarkers, solanezumab was able to induce a large and sustained increase of plasma Aβ_40_ and Aβ_42_, whereas in CSF it caused an increase of total Aβ and a decrease of free Aβ, indicating target engagement. There were no significant effects in CSF tau and p-tau levels. No differences were observed with respect to the placebo for whole-brain and hippocampal volume loss, as well as for amyloid-PET, although the sample set was small. Overall, these two phase III studies were considered negative [54]. However, a subsequent secondary analysis of efficacy on the pooled mild AD population of the two trials found a reduction of cognitive and functional decline, as indicated by significant changes in ADAS-Cog11 and 14, MMSE, and ADCS-i(instrumental)ADL, in solanezumab-treated patients with respect to those receiving the placebo [55]. Thus, a third randomised, double-blind, placebo-controlled, phase III trial (EXPEDITION 3) was conducted only on patients with mild AD (MMSE 20–26) and with amyloid deposition as assessed by PET or Aβ_42_ measurements in CSF [56]. The study enrolled 2197 patients, 1057 of whom were assigned to receive solanezumab at the dose of 400 mg every 4 weeks for 76 weeks. The primary outcome was the change of ADAS-Cog14 score, whereas secondary outcomes were changes in MMSE, ADCS-ADL, ADCS-iADL, CDR-SB, FAQ (Functional Activities Questionnaire), and iADRS (Integrated Alzheimer’s Disease Rating Scale), in all cases from baseline to 80 weeks. Approximately 86% of patients in the solanezumab group and 85% in the placebo group completed the study. Unfortunately, the results of this trial were negative, with solanezumab not showing robust beneficial effects both on primary and secondary outcomes, despite the observation that it was able to reduce free plasma Aβ levels by more than 90%.

A recent meta-analysis performed on the pooled data of the three EXPEDITION trials on a total of 3437 patients with mild AD (1728 randomised to placebo and 1709 randomised to solanezumab) reported a significant, but limited, slowing in cognitive and functional decline (ADAS-Cog14, ADCS-ADL, ADCS-iADL, CDR-SB, iADRS, and MMSE; range of slowing from 14 to 21%) at 80 weeks in patients treated with solanezumab [57]. 

Since accumulation of Aβ and tau begins more than a decade before the manifestation of clinical symptoms of cognitive impairment, an early therapeutic intervention should produce more evident beneficial effects or even halt the progression to dementia. On this basis, solanezumab was trialled on cognitively unimpaired older persons having elevated amyloid accumulation who are considered to represent an asymptomatic stage of AD (preclinical AD) and are at high risk of progressing to cognitive decline over 3–5 years. The A4 study was a randomised, double-blind, placebo-controlled phase III trial that enrolled preclinical AD individuals who were cognitively normal at baseline (CDR = 0; MMSE = 25–30; Wechsler Memory Scale Logical Memory Delayed Recall, LMDR = 6–18) and presented elevated amyloid levels, as assessed by amyloid-PET imaging using a quantitative method with a defined threshold [58]. At the end of the screening and randomisation, 578 persons were assigned to receive solanezumab (initially 400 mg, then increased to 1600 mg i.v. every 4 weeks for 240 weeks) and 591 to receive the placebo. The primary efficacy endpoint was the change in the PACC (Preclinical Alzheimer Cognitive Composite, including four components) score at 4.5 years, while secondary endpoints included changes in CFI (Cognitive Function Index), ADCS-ADL Prevention Questionnaire, and CDR-SB, with assessments performed at five time points after baseline. Also in this case, there were no significant differences in the change of the PACC score between solanezumab- and placebo-administered patients, with an unexpected greater decline in the solanezumab group. Although failure to reach significance in the primary endpoint did not allow the analysis of significance for the secondary endpoints, the results showed an unexpected worsening in the solanezumab group as compared with the placebo group. PET analyses showed that tau increased to the same extent in both groups, whereas solanezumab seemed to slow amyloid accumulation. 

#### 3.3.3. Gantenerumab

Gantenerumab is a fully human IgG1 anti-Aβ monoclonal antibody designed to promote the clearance of plaques by binding with high affinity to a conformational epitope present on Aβ fibrils. The antibody recognises both N-terminal and central regions of the peptide and induces its removal by Fc receptor-mediated microglial phagocytosis [59,60]. A recent in vitro study showed that gantenerumab preferentially binds Aβ fibrils over small and large protofibrils and has a low affinity for monomers [61]. Table 3 summarises the main results obtained with this antibody in phase III trials.

Gantenerumab was tested in the Scarlet RoAD (SR) trial, a randomised, double-blind, placebo-controlled phase III study on patients with prodromal AD, a symptomatic predementia phase of AD also referred to as mild cognitive impairment (MCI) due to AD [62]. A total of 799 patients met the eligibility criteria for prodromal AD according to the International Working Group criteria [63], showing biomarker evidence of amyloid pathology (CSF Aβ_42_ levels ≤ 600 ng/L) and absence of dementia diagnosis assessed with MMSE, CDR, CDR-SB, and FCSRT (Free and Cued Selective Reminding Test). Gantenerumab was administered subcutaneously (s.c.) at the doses of 105 mg (*APOE ε4* homozygotes) and 225 mg (*APOE ε4* heterozygotes and non-carriers) every 4 weeks for two years. The primary endpoint was the change in CDR-SB, while secondary cognitive, functional, and behavioural endpoints included changes in ADAS-Cog13, MMSE, CANTAB (Cambridge Neuropsychological Test Automated Battery), FCSRT, NPI-Q (Neuropsychiatric Inventory Questionnaire), and FAQ (Functional Activities Questionnaire).

The trial was halted after 4 years (December 2014) for futility, following the pre-planned interim analysis. At that time, 316 patients had completed the 2 years of the trial (108 placebo, 110 and 98 with gantenerumab 105 and 225 mg, respectively). The results from the exploratory efficacy analysis showed that no treatment effects were observed at 2 years for primary and secondary endpoints. Analysis of amyloid-PET, carried out on a small number of patients completing the 2-year treatment, revealed that the higher dose of gantenerumab was able to slightly reduce Aβ load from baseline by an average of 4.8%. As for CSF biomarkers, significant reductions of total/p-tau were observed in the gantenerumab groups, whereas there were no effects on Aβ_42_ levels. No differences were detected for any of the MRI volumetric measures at both gantenerumab doses. 

A further exploratory analysis was performed by applying the algorithm of an AD progression model to identify individuals predicted to be fast or slow progressors in the population of patients with prodromal AD [62,64]. The results of this analysis suggested a slowing of decline in the change of ADAS-Cog13, CANTAB, and MMSE but not of CDR-SB, in the gantenerumab-treated fast progressor subgroup, whereas no differences were observed in slow progressors. 

A second randomised, double-blind, placebo-controlled phase III trial, named Marguerite RoAD (MR), was also initiated in 2014 to evaluate the efficacy and safety of gantenerumab (105 or 225 mg s.c. every 4 weeks) in patients with mild AD, but recruitment was stopped following the futility analysis of the SR trial, while dosing continued. 

Since the results of the first trial suggested that higher doses of gantenerumab may have resulted in a more marked reduction of brain Aβ and in more relevant clinical effects, in 2015 both SR and MR trials were transformed in open-label extension (OLE) studies to evaluate the effects of a high dose of the antibody (1200 mg) achieved by titration regimens to minimise adverse events. An amyloid-PET sub-study interim analysis of the two OLE studies reported the results at 12 and 24 months for three different cohorts of patients with prodromal to moderate AD who participated in the double-blind period of the SR and MR trials: SR patients (SR), MR patients receiving the placebo (MR-DBP), and MR patients receiving the active drug (MR-DBA) [65]. The reduction in amyloid-PET in absolute centiloids was highly significant in all subgroups at both time points, with a reduction at 2 years of 64%, 77%, and 78% from baseline for SR, MR-DBA, and MR-DBP patients (39 completers), respectively. In addition, at the same time point, 51% of the patients showed Aβ levels below the positivity threshold. This open-label, non-placebo-controlled study was not designed to investigate clinical efficacy in terms of slowing disease progression. However, exploratory analyses of change from baseline to year 2 for some clinical endpoints (CDR-SB, ADAS-Cog11, and MMSE) were performed on completers and suggested a tendency for slower clinical decline at higher amyloid removal by gantenerumab [65]. The results of the amyloid-PET analysis were further confirmed by the results obtained at 3 years on 30 completers, demonstrating a continued reduction, with mean amyloid levels approaching zero centiloids for the three cohorts (SR, MR-DBP, and MR-DBA) and with the proportion of patients below the positivity threshold increasing to 80% [66].

In 2018, two other randomised, double-blind, placebo-controlled phase III parallel studies (GRADUATE I and II) began, with the aim of evaluating the efficacy and safety of gantenerumab (titrated over 9 months to a final dose of 520 mg every two weeks s.c. for 27 months) in patients with MCI due to AD and mild AD, collectively termed early AD (1965 participants randomised 1:1 to receive active drug or placebo). In November 2022, the sponsor announced that both studies did not meet the primary endpoint of slowing clinical decline as assessed with CDR-SB at 116 weeks. In fact, the results showed insignificant −0.31 and −0.19 changes from baseline, representing a relative reduction of 8% and 6% compared with the placebo in GRADUATE I and II, respectively [67]. The results on secondary endpoints were similar, showing only trends favouring gantenerumab. However, in these trials, gantenerumab significantly reduced Aβ load on average by approximately 23 (−24%) and 53 centiloids (−45%) vs. baseline, respectively, at years 1 and 2 [68].

#### 3.3.4. Solanezumab and Gantenerumab in DIAD

Dominantly Inherited Alzheimer’s Disease (DIAD) is the rare familial form of the disorder, accounting for <1% of all AD cases, in which the age of dementia manifestation can be largely predicted based on genetic mutations. In addition, the disease pathology manifests many years before symptom onset [69,70,71]. Therefore, in 2012, the DIAN-Trials Unit (DIAN-TU) was launched as a two-year phase II trial to evaluate the effects of solanezumab and gantenerumab on biomarkers in a population of asymptomatic or mildly symptomatic individuals with DIAD (Table 2 and Table 3). However, in 2015 the trial was transformed into a 4-year phase II/III trial to test whether the antibodies were able to prevent or slow disease progression [72]. In DIAN-TU, 52 individuals (60% asymptomatic) were randomised to receive solanezumab (dose increased from 400 to 1600 mg i.v. every 4 weeks), 52 (60% asymptomatic) to receive gantenerumab (dose increased from 225 to 1200 s.c., every 4 weeks), and 40 (55% asymptomatic) to receive a placebo for 4 years. A total of 105 persons completed the study (36 solanezumab, 39 gantenerumab, and 30 placebo). The primary endpoint was the measure of cognition using the DIAN Multivariate Cognitive End Point (DIAN-MCE), which included four different analyses, whereas secondary outcomes included CDR-SB and FAS (Functional Assessment Scale). Biomarker outcomes included amyloid-PET for gantenerumab, CSF total Aβ_42_, total tau, p-tau, and NfL (neurofilament, a marker of neurodegeneration) for solanezumab and gantenerumab. Overall, the analyses indicated no significant differences in cognitive decline between gantenerumab and placebo groups despite a significant between-group difference of 24.3% for Aβ deposition, 42.6% for increased CSF Aβ_42_ levels, and a 20.6% and 32.8% reduction for CSF total and p-tau, respectively. Also in the case of solanezumab, data analysis revealed no beneficial effects on cognitive measures or on biomarkers. Actually, the solanezumab group showed a faster cognitive decline.

**Table 2:** Phase III trials testing solanezumab.

Study Design and Duration	Study Population	Cognitive Primary Endpoint	CSF or Plasma Aβ	Amyloid PET	Volumetric MRI
Phase III, randomised, double-blind, placebo-controlled (EXPEDITION 1, 2) 80 weeks [54]	Mild to moderate AD	ADAS-Cog11 and ADCS-ADL Significant small effects for ADCS-ADL only in EXPEDITION 2 Significant small effects in a secondary analysis on the pooled mild AD-population [55]	Significant increase in plasma and CSF	No significant effects	No significant effects
Phase III, randomised, double-blind, placebo-controlled (EXPEDITION 3) 76 weeks [56]	Mild AD	ADAS-Cog11 No significant effects	Significant increase in plasma and CSF	No significant effects	No significant effects
Phase III, randomised, double-blind, placebo-controlled (A4) 240 weeks [58]	Preclinical AD	PACC No significant effects	ND	Smaller increase vs. placebo	No significant effects
Phase II/III, randomised, double-blind, placebo-controlled (DIAN-TU) 208 weeks [72]	Dominantly inherited AD	DIAN-MCE No significant effects	Significant increase in CSF	No significant effects	No significant effects

ND, not determined.

**Table 3:** Phase III trials testing gantenernumab.

Study Design and Duration	Study Population	Cognitive Primary Endpoint	CSF or Plasma Aβ	Amyloid PET	Volumetric MRI
Phase III, randomised, double-blind, placebo-controlled (Scarlet RoAD) 104 weeks Terminated early [62] Transformed in OLE	Prodromal AD	CDR-SB No significant effects	No significant effects in CSF	Slight reduction	No significant effects
Phase III, randomised, double-blind, placebo-controlled (Marguerite RoAD) 100 weeks Terminated early Transformed in OLE	Mild AD	___	___	___	___
Phase III, randomised, double-blind, placebo-controlled (GRADUATE I–II) 27 months Terminated early [67,68]	Early AD	CDR-SB No significant effects	___	Significant reduction (on average −44% at 2 years)	___
Phase II/III, randomised, double-blind, placebo-controlled (DIAN-TU) 208 weeks [72]	Dominantly inherited AD	DIAN-MCE No significant effects	Significant increase in CSF	Significant reduction (−24% at 4 years)	ND

ND, not determined.

#### 3.3.5. Crenezumab

Crenezumab is a humanised monoclonal immunoglobulin G4 antibody that binds different forms of Aβ, including monomers, oligomers, and fibrils, although it shows a higher affinity for oligomeric Aβ [73,74]. Crenezumab (60 mg/kg i.v., every 4 weeks for 100 weeks) was tested in two randomised, double-blind, placebo-controlled phase III studies, CREAD and CREAD2, on individuals with early AD with confirmed Aβ pathology (Table 4) [75]. In the CREAD trial, a total of 173 (88 placebo and 85 active drug) out of 813 patients completed the study before it was discontinued for futility, following a pr-planned interim analysis. In the CREAD2 trial, no participants (399 placebo and 407 crenezumab) completed the study before discontinuation. The primary endpoint was the change in CDR-SB from baseline to week 105. Secondary endpoints included changes in CDR, MMSE, ADAS-Cog11 and 13, ADCS-ADLS, ADCS-iADL, and NPI-Q, as well as some analyses on quality of life and caregiver burden. 

In CREAD, no significant differences were demonstrated in CDR-SB between crenezumab and the placebo at any time point investigated, and the difference in mean change from baseline to week 105 was −0.17, favouring the placebo. No treatment effect was observed also for secondary endpoints. In CREAD2, analysis of the smaller dataset found a mean change in CDR-SB from baseline to week 77 of 1.3, favouring crenezumab. No significant effects were reported for secondary outcomes. Also, there were no differences in individuals with prodromal AD (MCI due to AD) in comparison with patients with mild AD from baseline to week 105 in the pooled CREAD/CREAD2 dataset of CDR-SB and other secondary outcomes. In CREAD, crenezumab significantly increased Aβ_40_ and Aβ_42_ in plasma and CSF, but there were no significant differences in longitudinal changes in amyloid-PET, volumetric MRI, Aβ oligomers in CSF, and total and p-tau.

In 2013, a randomised, double-blind, placebo-controlled phase II study, named the API (Alzheimer’s Prevention Initiative) ADAD (Autosomal Dominant AD) Colombia Trial, was initiated to evaluate the safety and efficacy of crenezumab in a cognitively unimpaired Colombian family of individuals carrying the *PSEN1* E280A mutation and in non-carriers [76]. In this family, the median age of onset for fibrillar Aβ deposition was 28 years, whereas the onset age for MCI and dementia was 44 and 49 years, respectively [77,78,79]. This 5- to 8-year trial enrolled 252 individuals, with approximately two thirds (167) carrying the mutation randomised to receive crenezumab initially at the dose of 300 mg s.c. every 2 weeks. In 2015, the dose was increased to 720 mg every 2 weeks, and in 2019 to 60 mg/kg i.v., every two weeks. The primary outcome was the change in the API Composite Cognitive (APICC) test score (comprising elements from five different tests) from baseline to week 260. Secondary outcomes included time to progression to MCI or dementia due to AD, time to a CDR >0, change of CDR-SB, and change in amyloid-PET, cerebral glucose metabolism (FDG PET), volumetric MRI, CSF levels of Aβ, and total and p-tau. Exploratory measurements also included the Free and Cued Selective Reminding Test (FCRST), Functional Assessment Staging of Alzheimer’s Disease (FAST), Geriatric Depression Scale (GDS), Neuropsychiatric Inventory (NPI), Repeatable Battery for the Assessment of Neuropsychological Status (RBANS), and Subjective Memory Checklist (SMC). 

In June 2022, the sponsor announced that the trial did not demonstrate a statistically significant clinical benefit for co-primary, multiple secondary, and exploratory endpoints, although small numerical differences favouring crenezumab were observed [80]. More detailed results were presented at the Alzheimer’s Association International Conference (AAIC) in July–August 2022 and confirmed that differences in the cognitive primary and in all the clinical secondary endpoints, as well as biomarker outcomes, did not reach statistical significance, although there was a trend favouring crenezumab [81]. In fact, crenezumab-treated individuals declined more slowly on the primary endpoint APICC (23%) and in some secondary and exploratory outcomes (20% on FCRST, 8% on CDR, 9% on CDR-SB, and 44% on RBANS). Progression to MCI or dementia due to AD also showed some slowing down. However, all differences were insignificant. Similarly, differences in biomarkers showed a non-significant trend in favour of crenezumab. The only exceptions were CSF Aβ_40_ levels, which significantly increased, and Aβ_42_ levels, which were stable in crenezumab-treated patients while decreasing in controls.

**Table 4:** Phase II/III trials testing crenezumab.

Study Design and Duration	Study Population	Cognitive Primary Endpoint	CSF or Plasma Aβ	Amyloid PET	Volumetric MRI
Phase III, randomised, double-blind, placebo-controlled (CREAD, CREAD2) 100 weeks Terminated early [75]	Early AD	CDR-SB No significant effects	Significant increase in CSF and plasma	No significant effects	No significant effects
Phase II, randomised, double-blind, placebo-controlled (API ADAD Colombia) 5 to 8 years Terminated early [76,80,81]	Cognitively healthy Colombian family	APICC No significant effects	Stable levels/significant increase in CSF	No significant effects	No significant effects

#### 3.3.6. Aducanumab

Aducanumab is a human monoclonal antibody selectively targeting different forms of aggregated Aβ, which, like gantenerumab, shows preferred binding to fibrils over protofibrils and has low affinity for Aβ monomers [61,82]. This antibody was tested in two randomised, double-blind, placebo-controlled phase III trials, EMERGE and ENGAGE, which randomised 1643 and 1653 patients with early AD to placebo or to aducanumab, respectively (Table 5). Two doses of the antibody were i.v. administered to patients every 4 weeks for 76 weeks. The low dose was 3 mg/kg for *APOE ε4* carriers and 6 mg/kg for non-carriers; the high dose was 6 mg/kg for carriers and 10 mg/kg for non-carriers. In EMERGE, 874 patients completed the study (288 placebo, 291 low dose, and 295 high dose), whereas 938 completed ENGAGE (325 placebo, 325 low dose, and 288 high dose). The primary endpoint was the change in CDR-SB, secondary endpoints were changes in MMSE, ADAS-Cog13, and ADCS-iADL-MCI, and the tertiary endpoint was NPI-10. The endpoint measures were assessed at baseline and at weeks 26, 50, and 78. In subsets of patients, amyloid-PET, tau-PET, and biomarker (CSF Aβ_42_, tau, and p-tau levels) analyses were performed.

The story of these trials is very interesting. The two studies initiated in 2015 and, as announced by the sponsor, were terminated in March 2019 following a pre-specified interim analysis for futility on the pooled data from EMERGE and ENGAGE, which indicated the drug to be ineffective on primary endpoints [83]. Some months later (October 2019), a post-hoc analysis on a larger dataset showed that EMERGE had met its primary endpoint in the high-dose group, while ENGAGE did not, and the sponsor announced its intention to pursue regulatory approval for aducanumab [84]. As recently published, a slight significant difference of −0.39 in the CDR-SB mean change from baseline to week 78 was found in EMERGE in the high-dose aducanumab group in comparison with the placebo, indicating a relative 22% reduction in cognitive decline [85]. Significant differences for aducanumab vs. the placebo were found in the MMSE (0.6 points, 18%), ADAS-Cog13 (−1.4 points, 27%), and ADCS-ADL-MCI (−1.7, 44%) at week 78. As for biomarker analyses, it was shown that the high dose of aducanumab was able to significantly and markedly reduce amyloid-PET from baseline to week 78 by 71% and 59% in EMERGE and ENGAGE sub-studies, respectively. In addition, 48% of EMERGE patients and 31% of ENGAGE patients treated with the high aducanumab dose showed a PET score at week 78 that was equal to or below the threshold value for amyloid positivity. In addition, significant effects of aducanumab (mean change vs. baseline) were observed in EMERGE for CSF p-tau (approx. −23 and −17 pg/mL for high and low dose, respectively), total tau (approx. −130 and −90 pg/mL), and CSF Aβ_42_ levels (approx. 280 and 140 pg/mL). Significant effects were also found for CSF Aβ_42_ in ENGAGE, but only at the high aducanumab dose. On the other hand, aducanumab did not show beneficial effects on brain atrophy, according to volumetric MRI analyses. Surprisingly, it caused significant increases in ventricular volume at both doses and in both trials. 

In November 2020, the FDA Peripheral and Central Nervous System Drugs Advisory Committee voted on several questions regarding the results for aducanumab [86,87]. In particular, the Committee voted one yes, eight no, and two uncertain on the question as to whether the EMERGE study had provided strong evidence supporting the effectiveness of aducanumab for the treatment of AD. Nevertheless, in June 2021, the FDA approved aducanumab using an accelerated approval pathway based on the fact that aducanumab was the first drug directly targeting the underlying pathophysiology of AD, namely Aβ plaques, and that the decrease in those plaques as shown in the clinical trials is expected to lead to a reduction of clinical decline. Of note, three standing members of the FDA PCNS Drugs Advisory Committee resigned following the FDA accelerated approval of aducanumab [88]. The very limited results obtained in the aducanumab phase III trials and the FDA’s decision to approve aducanumab based on the reduction of Aβ plaques as a surrogate measure of clinical efficacy, a claim that lacks conclusive evidence, has fuelled an endless and controversial debate in the scientific community [89,90,91,92,93,94,95,96,97,98,99,100]. Lastly, in December 2021, the European Medicine Agency (EMA) recommended the refusal of marketing authorisation of aducanumab, since the link between Aβ plaque reduction and clinical efficacy had not been established, the results of the two studies (EMERGE and ENGAGE) were conflicting and did not show efficacy in treating patients with early AD [101]. The company requested a re-examination of EMA’s recommendation, but the application was withdrawn in April 2022 before the re-examination procedure had been completed [102].

#### 3.3.7. Lecanemab

Lecanemab is a humanised IgG1 antibody targeting soluble Aβ oligomers and showing some preference for fibrils over protofibrils [61,103]. The Clarity AD study, a randomised, double-blind, placebo-controlled trial, investigated lecanemab in early AD patients over 18 months (Table 5) [104]. Following randomisation, patients with MCI due to AD and with mild AD (early AD) were assigned to receive a placebo (897, with 757 completing the trial) or 10 mg/kg lecanemab (i.v.) every two weeks (898, with 729 completing the trial). The primary efficacy endpoint was the change in the CDR-SB score, whereas secondary endpoints were change in ADAS-Cog14, ADCOMS (AD Composite Score), ADCS-MCI-ADL, and amyloid-PET, all from baseline to month 18. Biomarkers in CSF (Aβ_40/42_, total tau, p-tau, neurogranin, and NfL) and plasma (Aβ_40/42_ ratio, total tau, p-tau, GFAP, and NfL) were also assessed. Results showed that lecanemab caused a significant, though small, difference of −0.45 points in the mean change of the primary endpoint CDR-SB score (27% relative effect). However, it has been highlighted that, with regard to the primary endpoint, lecanemab was less effective in women, who have twice the risk of AD compared to men, and in *APOE ε4* carriers, especially in homozygotes who showed increased decline [105]. As for the secondary endpoints, changes of −1.44 points for ADAS-Cog14, −0.05 points for ADCOMS, and 2 points for ADCS-MCI-ADL were found with respect to the placebo. 

Amyloid-PET analysis of a subgroup of patients (698) showed a marked mean change vs. baseline of −55.48 centiloids in the lecanemab group (71% reduction), leading to amyloid levels below the threshold for positivity (approx. 30 centiloids). Analysis of biomarkers in plasma and CSF all showed non-significant numerical improvements in comparison with the placebo, except for CSF NfL. 

Similarly, the hazard ratio for progression of the disease (worsening of the CDR global score) numerically favoured lecanemab. Following accelerated approval in January 2023, in July 2023, it was announced that lecanemab received the traditional approval for marketing by FDA [106]. Also in this case, there is debate as to whether the limited effects of lecanemab observed in the Clarity AD trial could be clinically meaningful in the real world [107,108,109,110,111,112,113,114,115,116,117,118].

#### 3.3.8. Donanemab

Donanemab is a humanised IgG1 antibody able to bind the pyroglutamyl E3 Aβ peptide (Aβ_p3–42_), present only in brain amyloid plaques, thus inducing their removal by microglial phagocytosis [119]. In July 2023, the results of the 76-week, randomised, double-bind, placebo-controlled trial TRAILBLAZER-ALZ 2, testing donanemab, were published (Table 5) [120]. The study randomised 1736 early AD patients with low/medium or high tau pathology to receive a placebo (876, 80% completing the study) or donanemab (860, 72% completing the study) i.v. every four weeks, at the initial dose of 700 mg for the first three administrations and 1400 mg thereafter for up to 76 weeks. The primary endpoint was the change in the iADRS score (range 0–144) from baseline to week 76 in the low/medium or in the combined (low/medium + high) tau populations. Secondary outcomes were CDR-SB, ADAS-Cog13, ADCS-iADL, and MMSE. Additional secondary outcomes were amyloid-PET reduction at week 76, percentage of patients with amyloid clearance (<24.1 centiloids) at weeks 24 and 76, and change in tau-PET and in volumetric MRI. The results showed that, in the low/medium tau population, there was a significant mean change difference of 3.25 in the iADRS score between the donanemab and placebo groups, indicating a relative 35.1% slowing of disease progression. In the combined tau population, the significant mean change difference was 2.92, representing a relative 22.3% slowing of progression. As for secondary outcomes, significant mean change differences were observed both in low/medium and combined tau populations (respectively, −0.67 and −0.7 for CDR-SB, 1.83 and 1.70 for ADCS-iADL, and −1.52 and −1.33 for ADAS-Cog13). According to the time-based analysis reported, disease progression in the low/medium population was delayed by donanemab by 4.36 months on iADRS and 7.53 months on CDR-SB over 18 months. In the case of amyloid-PET, donanemab was able to induce a huge reduction of Aβ load in both populations at week 76 (−85.5% and −83.7% from baseline in medium/low and combined populations, respectively), with 80.1% (low/medium) and 76.4% (combined) of patients reaching amyloid clearance at week 76. On the contrary, frontal p-tau PET did not show significant differences from the placebo group at week 76 in both populations, whereas a significant decrease was observed in plasma. Volumetric MRI analyses in both populations showed that donanemab caused a significant greater decrease in whole brain volume, a greater increase in ventricular volume, and a lesser decrease in hippocampal volume. 

Again, whether the beneficial changes can be clinically relevant in the heterogeneous population of patients with early AD in the real world is under debate [121,122,123,124].

**Table 5:** Phase III trial testing aducanumab, lecanemab and donanemab.

Study Design and Duration	Study Population	Cognitive Primary Endpoint	CSF or Plasma Aβ	Amyloid PET	Volumetric MRI
***Aducanumab*** Phase III, randomised, double-blind, placebo-controlled (EMERGE, ENGAGE) 76 weeks Terminated early [85]	Early AD	CDR-SB Significant effect only in EMERGE (−0.39 mean change vs. placebo)	Significant increase	Significant reduction (−71% EMERGE, −59% ENGAGE)	Significant increase in lateral ventricle volume
***Lecanemab*** Phase III, randomised, double-blind, placebo-controlled (Clarity AD) 18 months [104]	Early AD	CDR-SB Significant effect (−0.45 mean change vs. placebo)	No significant effects	Significant reduction (−71%)	___
***Donanemab*** Phase III, randomised, double-blind, placebo-controlled (TRAILBLAZER-ALZ 2) 76 weeks [120]	Early AD	iADRS Significant effect (2.92–3.25 mean change vs. placebo)	ND	Significant reduction (−85% on average)	Significant decrease in whole brain volume Significant increase in lateral ventricle volume

ND, not determined.

In conclusion, the results of the many clinical trials summarised above hardly demonstrate beyond any doubt that Aβ, in any form, is the etiological factor of Alzheimer’s disease, as indicated in the amyloid cascade hypothesis. Although some studies investigating the anti-Aβ immunotherapy effects on cognitive decline did not include amyloid-PET to evaluate brain Aβ load as a direct measure of efficacy in target engagement, this is not the case for other, more recent trials.

To summarise the phase III studies that evaluated cognition and amyloid-PET variations:

- In the SR-MR OLE trials, gantenerumab showed a non-significant modest trend for slowing disease progression in early AD patients, despite a decrease of brain Aβ levels of up to 78% and 51% of patients below amyloid positivity threshold at 2 years.
- In the GRADUATE I and II trials, gantenerumab caused a very modest, insignificant effect on cognition in early AD patients at two years, although a 44% decrease in brain Aβ levels was observed, and 27% of patients became amyloid-negative at the same time point.
- In the ENGAGE study in early AD patients, aducanumab induced almost no variation in the cognitive primary endpoint with respect to the placebo at 78 weeks, but caused a 60% reduction of brain Aβ load, with 31% of patients having an amyloid-PET score at or below the threshold for positivity.
- In the EMERGE study (with exactly the same design as ENGAGE), aducanumab was able to significantly affect the cognitive primary endpoint, but the effect was modest (−0.39 in the CDR-SB mean change vs. placebo), despite a 71% reduction of Aβ burden and 48% of patients at or below threshold for amyloid-PET positivity.
- In the Clarity AD trial, lecanemab produced a significant change in the cognitive primary endpoint (−0.45 in the CDR-SB mean change vs. placebo) at 18 months, but again the effect was small if compared to the 71% reduction in brain Aβ levels, with all patients below the threshold for positivity.
- In the TRAILBLAZER-ALZ 2 trial, in early AD patients, donanemab produced a dramatic, more than 80%, decrease in brain Aβ, with almost 80% of participants reaching amyloid clearance at week 76. On the other hand, its significant effect on the cognitive primary endpoint was still limited (on average, −0.7 in the CDR-SB mean change vs. placebo).

Therefore, these data suggest that Aβ plays a minor, and not a central, role in the pathophysiology of AD. 

Of note, the effects observed with aducanumab, lecanemab, and donanemab are similar to those obtained with acetylcholinesterase inhibitors, such as donepezil (−2.67 points on ADAS-Cog, 1.05 points on MMSE, and −0.53 points on CDR-SB, compared to placebo over 6 months) [125].

While a thorough discussion on the side effects of anti-Aβ antibodies is outside the scope of this article, it must be borne in mind that these immunotherapy strategies, showing modest efficacy, are associated with amyloid-related imaging abnormalities (ARIA), characterised by cerebral microhaemorrhages/haemosiderosis (ARIA-H) and oedema/effusion (ARIA-E), especially in *APOE ε4* carriers [126,127]. Although it is reported that most cases were asymptomatic and resolved with discontinuation of therapy, limited data are available on what could happen with continuation of antibody administration. Furthermore, a recent meta-analysis showed that different Aβ-lowering therapies can induce an accelerated brain volume loss in AD patients, with a major impact on ventricular enlargement by anti-Aβ antibodies (aducanumab, bapineuzumab, donanemab, and lecanemab), thus suggesting their potential to alter brain health in the long term [128].

## 4. The Aβ Loss-of-Function Hypothesis

Most studies focused on the Aβ gain of toxic function, underlying its key role in triggering the neurodegenerative/synaptotoxic processes in AD. However, it is now clear that this small peptide can play a variety of physiological roles in the central nervous system. As a matter of fact, BACE1, the enzyme driving APP amyloidogenic processing, is highly expressed in the normal brain, both at the mRNA and protein levels [129]. Also, Aβ peptides are physiologically produced in the brain of mammals, as demonstrated by their presence in the extracellular space of mice through in vivo intracerebral microdialysis, or in the CSF of healthy, cognitively unimpaired individuals [130,131,132,133].

Some of the first preclinical evidence for the physiological roles of Aβ was already published in the late 1980s/early 1990s, when it was shown that low concentrations of the peptide exerted neurotrophic effects on cultured hippocampal neurons [134,135]. Indeed, it was later found that the reduction of Aβ levels, obtained with secretase inhibitors or with its immunodepletion, induced neuronal cell death, which was prevented by application of exogenous Aβ [136]. In line with this, the addition of exogenous monomeric Aβ was able to rescue neurons from insulin-deprivation- or excitotoxicity-induced cell death, further confirming the neuroprotective potential of this peptide [137].

Moreover, Aβ was shown to possess neurogenic effects [138], increase the density of total dendritic spines in organotypic hippocampal slices [139], promote oligodendrocyte differentiation and survival, and enhance remyelination in organotypic cerebellar slices [140].

Interestingly, Aβ could exert also protective effects after CNS injury, since it was reported that BACE1 deletion worsened cognitive and motor functions in experimental models of traumatic brain injury [141] and spinal cord injury [142]. Furthermore, it has been proposed that Aβ serves important antioxidant functions in the brain, especially by means of its metal-binding properties, and that its increase under different pathological conditions with oxidative stress components could represent the attempt of a neuroprotective response [143,144].

At the level of neurotransmission, evidence has accumulated showing that Aβ is an endogenous regulator of release probability, controls neuronal excitability, and interacts with different neurotransmitter systems at the presynaptic level [145,146].

One of the most striking findings is the physiological role of the amyloid peptide in memory, which started to emerge in the mid 1990s, when exogenous Aβ was shown to enhance hippocampal long-term potentiation (LTP), the synaptic plasticity phenomenon representing the electrophysiological correlate of memory formation/consolidation [147]. This observation was confirmed and extended by several studies demonstrating that low concentrations of exogenous Aβ enhanced hippocampal LTP and improved hippocampal-dependent memory (both effects mediated by α7-nicotinic receptors), whereas its immuno-mediated depletion abrogated LTP and induced significant cognitive impairments, which were rescued by physiological concentrations of the amyloid peptide [148,149,150,151]. In addition, it was found that physiological Aβ production is enhanced by both cAMP and cGMP, although through different mechanisms, and that the peptide is necessary for both nucleotides to trigger LTP and memory formation [152,153,154,155]. Indirect evidence of Aβ involvement in memory was also provided by studies showing that BACE1 knocking out or its pharmacological inhibition caused LTP alterations and cognitive deficits in mice [156,157]. 

Furthermore, an in vitro study evaluating the effects of 138 mutations in human PSEN1 found that 90% of them decreased, and not increased, the production of Aβ_40_ and Aβ_42_ [158]. 

Based on those findings, the loss of function (LOF), as opposed to the gain of toxic functions (GOF) proposed by the ACH, has been hypothesised as one of the possible pathophysiological mechanisms of AD due to the decreased availability of physiologically relevant forms of Aβ [159,160,161,162,163,164,165]. As a matter of fact, support of the LOF hypothesis comes from a recent preclinical study demonstrating that the in vivo intrahippocampal administration of the N-Aβcore (a synthetic peptide comprising amino acids 10–15 of the N-terminal region of Aβ) to 5xFAD APP/PS1 transgenic mice (a widely used model for familial AD) reversed the dramatic reduction of LTP as well as the enhancement of LTD observed in hippocampal slices of untreated controls [166]. The N-Aβcore also significantly reduced astrogliosis and microgliosis in ex vivo organotypic coronal brain slices obtained from 7-month-old 5xFAD mice, thus resulting in decreased neuronal loss [167]. Moreover, intracerebroventricular administration of human Aβ_42_ improved the impaired hippocampal LTP in APP/PS1/Tau triple transgenic AD mice, an effect that was associated with the amelioration of cognitive deficits in the Y maze spontaneous alternation test and in the object-location task [168]. 

However, what Aβ form(s) play key physiological roles and whose LOF can participate in the pathophysiology of AD has yet to be established, with some lines of evidence indicating the monomeric peptide and others pointing to oligomers [160,163,166,168,169,170,171].

In line with the LOF hypothesis, a cross-sectional study on 598 amyloid-positive individuals of the Alzheimer’s Disease Neuroimaging Initiative (ADNI) cohort reported that CSF soluble Aβ_42_ levels were higher in those amyloid-PET-positive participants performing better in neuropsychological evaluation for memory and executive functions [172]. Specifically, CSF levels of Aβ_42_ were significantly higher, cognitive performance was significantly better, and hippocampal volume was significantly greater in cognitively normal individuals with amyloid-PET positivity (155) than in those with MCI (271) who, in turn, had significantly higher peptide levels, better cognitive performance, and larger hippocampal volume than patients with AD (172). Moreover, the analysis of conversion from MCI to AD (103 subjects) suggested that the decrease in soluble Aβ_42_ was more critical than an equivalent elevation in amyloid brain load, thus supporting the LOF hypothesis. Intriguingly, a retrospective longitudinal study on 108 amyloid-PET-positive subjects of the DIAN cohort (patients at risk for carrying a mutation responsible for ADAD; see above) revealed that the risk of CDR progression (i.e., worsening of cognition) was significantly reduced in those individuals with higher CSF Aβ_42_ levels, which were also associated with a decreased hazard of time to first CDR progression, even at high amyloid-PET values [173]. 

Of course, the LOF and GOF hypotheses may not be mutually exclusive in that the generation of toxic Aβ oligomers could reduce the monomeric pool of the peptide to a level that jeopardises several important physiological cellular functions. However, given the now generally accepted multifactorial nature of AD, in which dysfunctions of many pathways occur also independently of Aβ [174,175,176,177,178], it is unlikely that Aβ GOF or LOF, or both, can be the sole triggers precipitating the brain in the neurodegenerative processes characterising AD. 

## 5. Concluding Remarks

Over last decade, the amyloid cascade hypothesis has been increasingly subjected to a growing number of criticisms confuting the etiopathogenetic role of amyloid-beta, especially considering the negative results of most of the numerous clinical trials that tested the efficacy of anti-Aβ antibodies [34,161,179,180,181,182,183,184]. Paradoxically, the positive results recently obtained with aducanumab, lecanemab, and donanemab in early AD seem to support this view, as they showed modest beneficial effects on cognition despite marked effects on the clearance of the amyloid peptide from the brain. This is not to say that Aβ is not involved in the evolution of AD, but it is one of the participants in the pathological processes of the disease and not the culprit we were looking for. Moreover, the key physiological roles of this peptide in brain processes uncovered to date indicate that the use of anti-Aβ antibodies in AD needs to be cautiously evaluated, especially in the preclinical phase of the disease. 

Finally, like other neurodegenerative disorders, AD is now considered a multifactorial pathology and, therefore, greater efforts are required to identify and characterise in depth other druggable pathocascades for the development of multitarget therapeutic strategies able to halt or effectively modify this devastating disease.

## Data Availability

Not applicable.
